# Virulence and biofilms as promising targets in developing antipathogenic drugs against candidiasis

**DOI:** 10.2144/fsoa-2019-0027

**Published:** 2020-02-03

**Authors:** Mohd Sajjad Ahmad Khan, Fatimah Alshehrei, Saleh Bakheet Al-Ghamdi, Majid Abdullah Bamaga, Abdullah Safar Al-Thubiani, Mohammad Zubair Alam

**Affiliations:** 1 Department of Basic Sciences, Imam Abdulrahman Bin Faisal University, Dammam, Saudi Arabia; 2 Department of Biology, Faculty of Applied Science, Umm Al-Qura University, Makkah, Saudi Arabia; 3 Department of Biology, Faculty of Science, Al-Baha University, Baha, Saudi Arabia; 4 Department of Medical Laboratory Sciences, Fakeeh College for Medical Sciences, Jeddah, Saudi Arabia; 5 King Fahd Medical Research Center, King Abdulaziz University, Jeddah, Saudi Arabia

**Keywords:** antifungal, antipathogenic, biofilm, *Candida albicans*, candidiasis, drug discovery, hydrolytic enzymes, metabolic pathways, morphogenesis, virulence factors

## Abstract

*Candida albicans* has remained the main etiological agent of candidiasis, challenges clinicians with high mortality and morbidity. The emergence of resistance to antifungal drugs, toxicity and lower efficacy have all contributed to an urgent need to develop alternative drugs aiming at novel targets in *C. albicans*. Targeting the production of virulence factors, which are essential processes for infectious agents, represents an attractive substitute for the development of newer anti-infectives. The present review highlights the recent developments made in the understanding of the pathogenicity of *C. albicans*. Production of hydrolytic enzymes, morphogenesis and biofilm formation, along with their molecular and metabolic regulation in *Candida* are discussed with regard to the development of novel antipathogenic drugs against candidiasis.

## Lay abstract

Over the last decade, candidiasis has remained a major problematic disease worldwide. In spite of the existence of many antifungal drugs, the treatment of such diseases has still remained unsuccessful due to drug inefficacy. Therefore, there is a need to discover antifungals with different modes of action, such as antipathogenic drugs against *Candida albicans*. Here, we describe how various types of virulence factors such as proteinase, phospholipase, hemolysin, adhesion, morphogenesis and biofilm formation, could be targeted to develop novel therapeutics. We can inhibit production of these virulence factors by controlling their molecular/metabolic regulation.

## Graphical abstract

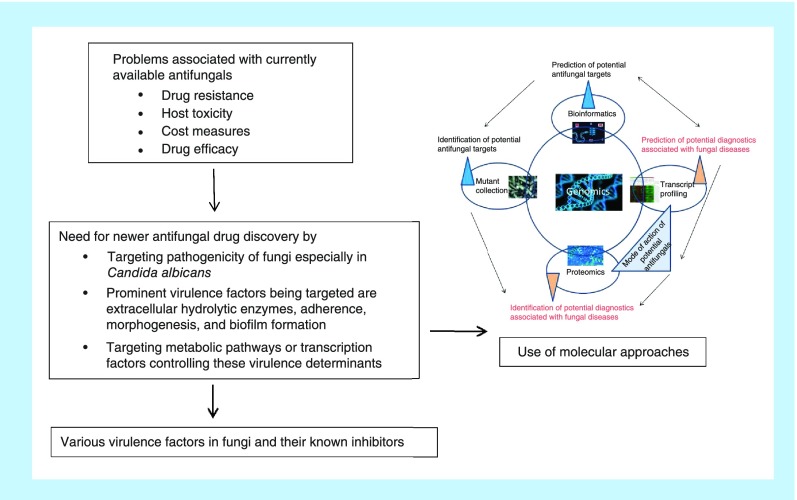

Candidiasis is an opportunistic infection that may be acute, subacute or chronic and often results in life threatening mycoses. Healthy individuals encounter superficial infections such as vulvovaginal candidiasis, candiduria, onychomycosis and oropharyngeal candidiasis. Conversely, immunocompromised patients develop invasive systemic candidiasis such as candidemia and organs infection, especially those of brain, kidneys and eyes [1,2]. The most common *Candida* species (spp.) that cause candidiasis are *C. albicans*, *C. dubliniensis*, *C. glabrata*, *C. krusei*, *C. parapsilosis* and *C. tropicalis* [2,3]. Among these, *C. albicans* dominates in causing infections of oral, genital and cutaneous sites, including patients in intensive care units with indwelling devices, as well as those who have undergone bone marrow transplantation and individuals treated improperly with broad spectrum antibiotics or corticosteroids [3,4]. A significant number of women witness vulvovaginal candidiasis at least once in their lifetime [5–7]. *Candida* spp. are the commonly isolated pathogens in nosocomial infections and are ranked as the fourth major causative agents of systemic infections, with a mortality rate of 50% [6,8,9].

Antifungal drugs used for invasive fungal infections are categorized as polyenes, azoles and echinocandins [10]. The careful use of these available antifungal agents and the management of underlying diseases have led to success in the treatment of invasive fungal diseases [11]. However, the emergence of drug-resistant strains and drug toxicity have indicated the need for a continuous search for novel antifungal drugs. In a blatant contrast with antibacterial drugs, the existing armaments of antifungal drugs are extremely diminutive. Moreover, the advancements in antifungal drug discovery programmes are slower than those for antibacterial drug discovery [12,13]. The currently available antifungal drugs target fungal growth. The drug that targets cell growth enforces a higher level of selective pressure, which results in the emergence of antibiotic-resistant strains [14]. Moreover, both host cells and fungi are eukaryotic and therefore share common physiological processes. This is also one of the main reasons for the noticeable host-toxicity of some of the existing antifungals. Hence, it is difficult to identify a drug with pathogen-specific targets during drug discovery and development programmes [12,15].

An alternative approach to antifungal drug development is to target pathogen-specific virulence factors. It is a quite effective strategy, as it maintains the host microflora with reduced cellular toxicity [14]. Also, considering the immunological aspects, the treatment of hosts with an antivirulence compound would result in a scenario similar to the use of live attenuated vaccines [12]. Therefore, understanding the infection biology of a pathogen is mandatory in recognizing new drug targets. In this review, we have highlighted some of the recent developments made in understanding how virulence traits including biofilm formation regulated at metabolic and molecular levels and, how this could be exploited as promising anticandidal drug targets.

## Current antifungal drug therapy: targeting cell growth & its challenges

**Figure 1.**
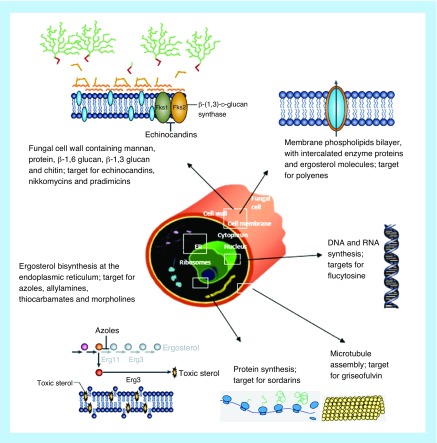
Antifungal drugs and their targets. The main classes of antifungal drugs that are in clinical use and how they exert their effects on the fungal cell (adapted from [15,18,181]).

Antifungal agents currently in use belong to seven classes of drugs: polyenes, azoles, allylamines, candins, morpholines, thiocarbamates and pyrimidine analogues [16]. All of these agents target cell growth and their mechanism of action are represented by inhibition of ergosterol biosynthesis; inhibition of DNA or RNA synthesis; and inhibition of glucan, chitin or mannan synthesis [17]. The principal targets of these antifungal drugs are varied and are depicted in Figure 1 as well as listed in Table 1.

**Table 1.** Antifungal agents: activities, mechanism of action and resistance against fungal pathogens.

Antifungal drugs	Activity spectrum	Mechanism of action	Mechanism of resistance	Ref.
**Polyenes**				
Amphotericin B	Broad activity against *Candida* spp (except *C. lusitaniae*), *Cryptococus neoformans* and filamentous fungi (except *Aspergillus* spp. *A. terreus* and *A. flavus*)	Binding to ergosterol and destabilization of cell membrane functions	Decreased access of AMB to drug target in fungal membrane, altered membrane ergosterol content and reduced intercalation, increased cell wall rigidity, sequesteration of fungi to lysosomes, enhanced catalase activity	[15,30]
Lipid formulations		Interaction with ergosterol, intercalation of fungal membrane that leads to increased permeability to univalent and divalent cations and cell death		
Liposomal nystatin		Alternative mechanism of action through oxidation of fungal membrane	Decreased oxidative damage (a) over expression of catalases and superoxide dismutases of *A. fumigatus*, (b) anaerobic environment	[15,16]
**Azoles**				
Miconazole, Ketoconazole, Clotrimazole, Fluconazole	Active against *Candida* spp, *Cryptococcus* spp, less active against *C. glabrata*, no activity against *C. krusei* and filamentous fungi	Interaction with cytochrome P-450 and inhibition of C-14 demethylation of Lanosterol (ERG11), causes ergosterol depletion and accumulation of toxic and aberrant sterols in membrane leading to perturbation of fungal cell membrane	Enhanced efflux by upregulation of multi-drug transporter genes (*CDR*, *MDR*), over expression of target enzyme, decreased affinity to the binding site, target alterations by occurrence of mutations (ERG3), alteration of specific steps in the ergosterol biosynthetic pathway, altered drug uptake, exogenous cholesterol import	[15,16,18]
Itraconazole	Like fluconazole but enhanced activity against filamentous fungi and other yeasts			
Voriconazole, Posaconazole, Ravuconazloe	Like fluconazole but enhanced activity against filamentous fungi including *Aspergillus* and *Fusarium* spp. Active against *C. krusei*, *C. glabrata* and *Candida* isolates with acquired azole resistance			
**Allylamine**				
Terbinafine	Active against most of dermatophytes, but poorly active against *Candida* spp	Inhibition of squalene epoxidase (ERG1), with subsequent ergosterol depletion and accumulation of toxic sterol intermediates	Increased drug efflux (CDR1, CDR2), over expression of target site (ERG1), over expression of salicylate mono-oxygenase (drug degradation)	[18]
**Morpholine**				
Amorolfine	Active against most of dermatophytes, but poorly active against *Candida* spp	Inhibition of sterol Δ^14^ reductase and Δ^7,8^ isomerase	Over expression of ERG24, *ERG4* genes	[18,30]
**Nucleoside analogue**				
5-Fluorocytosine (5FC)	Active against *Candida* spp and *Cryptococcus* spp.	Impairment of nucleic acid biosynthesis by formation of toxic fluorinated pyrimidine antimetabolites	Decreased uptake of 5-FC, decreased formation of toxic antimetabolites, defect in cytosine permease	[15,16,18]
**Echinocandins**				
Caspofungin Micafungin Anidulafungin	Active against *Candida* spp., moderately active against *Aspergillus* spp, poorly active against *C. neoformans*	Inhibition of the cell wall synthesis enzyme β-1,3-glucan synthase, leading to susceptibility of fungal cell to osmotic lysis	Up regulation of homeostatic stress-response pathways (HSP90; calcineurin), over expression of target site, up regulation of genes encoding for β–glucan synthetase (*FKS1* genes), over expression of genes related to transport of cell wall components	[15,16,18,30]

AMB: Amphotericin B.

The host toxicity and the rapid emergence of resistant strains are the main problems associated with these antifungal drugs, though low potency, poor solubility and limited or inconvenient dosage forms may also be accounted [19]. Amphotericin B fungal cell toxicity is due to its higher affinity toward ergosterol, resulting in pore formation and leakage of cytoplasmic material. However, it has been considered as toxic to hosts as well because it also has shown sufficient affinity toward cholesterol in the host cell membrane, and thereby affecting permeability of renal tubules [19–21]. 5-Fluorocytosine is known to obstruct DNA synthesis and may lead to bone marrow toxicity, leukopenia and imbalance of liver enzymes [22]. Nonetheless, globally, the most commonly prescribed antifungal drug is fluconazole because it is considered as the safest. However, its fungistatic nature has led to the development of drug-resistance and higher doses are reported to be hepatotoxic [23,24]. The echinocandins and the nikkomycins target the fungal cell wall by inhibiting glucan and chitin synthesis respectively. Such targets are not present in a mammalian cell; therefore, these drugs are less toxic to the host cells. Hence, these classes of antifungals have become attractive for use in humans, but are very expensive [18,25,26]. Recent reports have established increased drug resistance against these classes of drugs as well [13].

The evolution of antifungal drug resistance and its epidemiological spread is a key issue in the management of fungal diseases [27]. Molecular mechanisms of drug resistance in fungi are summarized in Table 1.

The formation of biofilms by many pathogenic *Candida* spp. are reported to confer drug resistance up to 1000-fold higher than the majority of antifungal drugs, particularly azoles and polyenes [16,28]. The mechanism of drug resistance shown by biofilms involves restricted infiltration of drugs inside the exopolymer matrix; limitations with the availability of nutrients, resulting in a decreased growth rate; overexpression of resistance genes, especially efflux pumps; and the existence of persister cells [29,30].

## Antifungal drug discovery: processes & new approaches

Antifungal drug development relies on the use of *Saccharomyces cerevisiae* as a successful model of a eukaryotic organism because it shares many molecular and metabolic processes similar to humans. This model is also being used to predict the potential efficacy or toxicity of drug candidates such as small molecule compounds [10,31]. The most common tactic to discover active small molecules as antifungal agents, is to screen libraries of compounds of natural or synthetic origin, which inhibit the growth of selected fungi [10]. Many researchers have attempted to develop newer chemotherapeutic drugs against fungi. Babazadeh-Qazijahani *et al.* [32] synhthesized a series of imidazolylchromanone oximes containing phenoxyethyl ether moiety similar to omoconazole. These copmpounds exhibited potent activity against *Cryptococcus gattii*. In search of newer kinds of azole drugs, Hashemi *et al.* [33] designed a series of triazole alcohols in which one of the 1,2,4-triazol-1-yl group in fluconazole structure has been replaced with 4-amino-5-aryl-3-mercapto-1,2,4-triazole motif. Furthermore, they removed the amino group from the structure to obtain 5-aryl-3-mercapto-1,2,4-triazole derivatives. In their study, the majority of the compounds were shown to be potential antifungal candidates even greater than fluconazole against *Candida* spp.

Coining of compounds exhibiting novel targets against the fungal cell and, the advanced drug-delivery approaches are of the utmost importance when developing new antifungal therapy. Components of the cell membrane and cell wall, various virulence factors including biofilm formation and other putative genes, have been considered as prime targets. As far as fungal cell wall is concerned, it is a vital structure for fungi but missing in mammalian cells, therefore it can be a target of high priority. Other promising targets for developing new antifungal drugs are efflux pumps, proton ATPase, metabolic pathways of nucleic acid, signal transduction and cell cycle [34].

## Targeting virulence factors to discover novel antifungal drugs

In the past few years, virulence factors of fungi and their inhibitors have been considerably investigated and characterized, which has led to the development of new alternatives for antifungal therapeutics [35]. Many different types of determinants such as genes (e.g., adhesins) and gene products (e.g. secreted enzymes) are considered as virulence factors that are involved in the host–microbe relationship, leading to superficial as well as invasive infections in humans [36]. Virulence factors facilitate adherence, infiltration and spread in host tissues, even at elevated temperatures. These factors also help pathogens to tolerate the hosts adaptive immunity for example, the evasion from phagocytes and the complement-mediated pathway. Additionally, nutritional, metabolic and necrotic factors or even morphology variations, including phenotype switching and biofilm formation, could be considered as virulence factors [37]. Virulent strains of *albicans* and *non-albicans Candida* specifically, *C. albicans, C. glabrata, C. auris* and *C. haemulonii*, are reported to potentially cause invasive fungal infections even in immunocompetent individuals [38].

Targeting virulence in developing antifungals has the edge over other strategies since it enumerates the number of potential targets that are needed for discovery of newer drug candidates; it maintains natural host microflora, which is of utmost importance especially for *C. albicans*; it exerts a low level of selective pressure, therefore it minimizes the development of antibiotic resistance [12,14]. Here, we have summarized some of the potential virulence factors in *C. albicans* and how they are being targeted by some of the compounds exhibiting antivirulence activity that could be a lead in drug discovery for antifungal drug development. Table 2 describes some of the compounds discovered as antivirulence and antibiofilm agents, which are expected to be drug leads.

**Table 2.** Occurrence of various virulence factors in fungi, their role and known inhibitors.

Virulence factors	Organisms	Role	Inhibitors	Ref.
Proteinases	*C. albicans*	Hydrolytic enzyme	Pepstatin A, saquinavir, indinavir Human domain antibodies	[175,176]
Phospholipases	*C. albicans, C. neoformans, Aspergillus flavus*	Hydrolytic enzyme	Alexidine dihydrochloride, 1,12 bis-(tributylphosphonium)-dodecane dibromide	[177]
Haemolysin	*C. albicans*	Hydrolytic enzyme	Cationic lipo-benzamide compound C9M	[178]
Candidalysin	*C. albicans*	Hydrolytic enzyme	Cis-2-dodecenoic acid	[179]
Elastase	*Trichophyton mentagrophytes, Candida* spp	Hydrolytic enzyme	Aliphatic aldehydes	[65]
Glyoxilate cycle	*C. albicans*	Metabolic pathways	Caffeic acid, rosmarinic acid and apigenin	[142]
Inositol phosphoryl ceramide synthase (IPC1)	*C. neoformans, Candida* spp, *Aspergillus* spp	Metabolic pathways	Aureobasidin A, khafrefungin	[180]
Isocitratelyase (ICL)	*C. albicans*	Metabolic pathways	3-nitropropionate, 3-bromopyruvate, mycenon, mohangamide A and mohangamide B	[153,154]
Target of Rapamycin (TOR) signaling pathway	*Saccharomyces cerevisae*	Metabolic pathways	Small molecule CID 3528206	[181]
Calcineurin	*C. albicans, C. neoformans*	Metabolic pathways	Tacrolimus, cyclosporin A	[150]
Hyphal formation	*Candida* spp, *C. neoformans*	Morphogenesis	Saponins	[88]
Adhesion, morphogenesis, biofilm	*C. albicans*	Ras1-cAMP-Efg1 pathway	Magnolol and honokiol	[182]
Biofilm	*Candida* spp	Drug-resistance	Farnesol, Diazaspiro-decane structural analogs, cationic lipo-benzamide compound C9M	[101,113,179]

### Extracellular hydrolytic enzymes

Extracellular hydrolytic enzymes assist fungi to rupture and enter host tissues [39]. Therefore, they are expected to be potential virulence factors. Some of these important enzymes are lipases, phospholipases and proteinases. Several studies have reported a reduction in virulence of *Candida* spp. due to the absence or reduced expression of these hydrolytic enzymes. These enzymes also help *Candida* cells to undergo morphological transitions, colonization and penetration of host tissues [39–41].

Phospholipases facilitate *Candida* cells in the invasion of host tissues by hydrolyzing ester linkages of glycophospholipids. It is evident from a study conducted by Ibrahim *et al*., that invasive strains of *C. albicans* produce a higher amount of phospholipases compared with noninvasive strains [42]. In *C. albicans*, four types of phospholipases are categorized, phospholipase A, B, C and D, depending upon the ability of the enzyme to cleave a specific ester bond [43]. Many researchers have observed that the invasiveness of *Candida* cells toward the epithelial tissues is facilitated by the higher production of phospholipases [43–46].

The secreted aspartyl proteinases (SAPs) play central role in *Candida* pathogenicity. A family of ten SAPs (Sap proteins) fulfill a number of specialized functions during the infective process. SAPs digest hemoglobin to acquire nutrition for *Candida* cells. They destroy the host cell membrane by hydrolyzing many tissue proteins such as albumin, collagen, cystatin A, keratin, laminin and fibronectin, to facilitate adhesion and tissue invasion. SAPs also digest cells and molecules of the host immune system such as IL-1β, immunoglobulin A and mucinand salivary lactoferin, to avoid or resist antimicrobial attack by the host [47]. Several workers have reported that the production of SAPs is accompanied by other factors such as adherence, hyphal formation and phenotype switching, which enhances pathogenicity [47–49].

Hemolysin, a mannoprotein attached to the cell surface, enables *C. albicans* to exploit iron from host protein [50]. Iron is an indispensable cofactor for several proteins and is a prerequisite for various metabolic processes such as,cellular respiration and DNA synthesis. Hence, it is considered as a crucial virulence factor in *C. albicans*. [51]. It has been reported that colonization and the spread of fungal cells are more pronounced if iron was easily available in an ample amount to the fungus [52]. *C. albicans* can also acquire iron from host ferritin via hyphal-associated adhesin and invasin Als3 [53].

A newly identified peptide toxin, candidalysin, which is derived from the product of the *ECE1* gene, is also categorized as an important virulence factor of *C. albicans* because it damages the host cell plasma membrane [54]. Candidalysin is found to stimulate the transcription factor c-Fos of activating protein 1 (AP-1) (via p38–mitogen-activated protein kinase [MAPK]) and the MAPK phosphatase MKP1 (via extracellular signal-regulated kinases 1 and 2 [ERK1/2]–MAPK), which trigger and regulate proinflammatory cytokine responses, respectively [55]. During pathogenesis, candidalysin triggers NLRP3 inflammasome-dependent caspase-1 activation via potassium efflux and acts as the main facilitator of inflammasome-independent cytolysis of macrophages and dendritic cells in the host [56].

#### Anti-secreted hydrolytic enzymes

A very important group of antipathogenic drugs are inhibitors of protease in fungi. It has been found that under *in vitro* conditions, some of the potential HIV protease inhibitors such as saquinavir and indinavir have dose-dependent inhibitory effects on SAPs [57,58]. SAPs of *C. albicans*, as well as HIV proteinases, belong to the group of aspartic proteinases and are inhibited by pepstatin A. The inhibitory effects of saquinavir and indinavir on SAPs *in vitro* are similar to pepstatin A. The study by Ollert *et al*. reported that pepstatin A retards adhesion of *Candida* to the host cells [59]. Therefore, the development of saquinavir and indinavir as potential antipathogenic drugs against *Candida* could be justified [35].

Another virulence factor that significantly contributes to the invasiveness of *C. albicans* during infection is phospholipase. Ganendren *et al.* [60] reported that commercially available compounds, like alexidine dihydrochloride and 1,12, bis-(tributylphosphonium)-dodecane dibromide with structural similarities to phospholipid substrates, had relatively broad antifungal activities under *in vitro* conditions against *C. albicans, Cryptococcus neoformans* and *Aspergillus flavus*. Orlistat is a saturated derivative of lipstatin, a powerful natural inhibitor of pancreatic lipases, first isolated from the bacterium *Streptomyces toxytricini*. It was prepared to treat obesity [61]. It has been shown to possess ten binding possibilities against the active site of *Candida rugose* lipase as observed in a docking study [62]. Other lipase inhibitors such as quinine and ebelactone B have been reported to retard the growth of *Candida* spp. [63,64].

Some aliphatic aldehyde compounds obtained from olive fruit extracts such as hexanal, nonanal, (E)-2-hexenal, (E)-2-heptenal, (E)-2-octenal and (E)-2-nonenal, have been reported to possess anti-elastase activities against dermatophytes and *C. albicans* [65]. Some studies have highlighted the efficacy of natural products such as oils of *Carum copticum*, *Cinnamomum verum*, *Syzygium aromaticum*, *Thymus vulgaris* and their active compounds, cinnamaldehyde and eugenol, at subinhibitory concentration, in inhibiting the production of proteinaes, hemolysin and biofilm formation in multi-drug resistant strains of *C. albicans* [66,67]. El Zawawy *et al.* [68] evaluated the efficacy of *Pluchea dioscoridis* leaf extract on growth, morphogenesis and virulence gene expression of *Candida albicans* and, observed greater than 70% decrease in expression of phospholipase, proteinase and hemolysin genes. The compounds inhibiting extracellular hydrolytic enzymes could be attractive molecules for anticandidal drug discovery.

### Morphogenesis

Morphogenesis in *C. albicans* is defined as a switch from yeast form to hyphal form. These morphological transitions are reversible and occur during growth. These kinds of physical plasticity have been considered to ease pathogenicity [31,69]. Morphogenetic transitions in fungi occur in reaction to an external stimulus, which may be encountered in a human host such as body temperature, pH, serum, nutrient and oxygen supply and certain hormones [69]. Yeast cells are capable of disseminating more efficiently than filamentous forms, which are rather well adapted for penetration and damaging the tissues [31,70]. Morphological transitions in *Candida* cells lead to the successful establishment of disease and further progression in the form of biofilms. Therefore, these factors are well accepted as contributors to pathogenicity in *C. albicans* [12,31].

#### Antimorphogenesis

To understand the molecular mechanisms involved in morphogenetic conversions in fungi, many researchers have investigated activities of signaling pathways, along with key transcriptional regulators [31]. Here, we have briefly summarized studies conducted to explore antimorphogenetic approaches to discover antifungal compounds.

##### Targeting filamentation in *C. albicans*

Filamentation is regulated in coordination with other virulence factors associated with cellular morphology [71]. Studies have evidenced that filamentation plays an active role in the progression of infection and could be targeted to obtain newer antifungal agents [12,70,72]. Additionally, immune cells respond differently to yeast and hyphal cells. In general, a protective host response is elicited against the exposure to yeast forms, whereas a nonprotective host response is elicited against filamentous forms [73,74]. Therefore, one very fascinating possibility is that anti-filamentation drug compounds could also benefit the human host by tempering immune responses [12].

##### Controlling multiple signaling pathways of filamentation in *C. albicans*

The process of filamentation in *C. albicans* is tightly regulated either positively or negatively, through manifold signaling pathways congregated on either the same, or different transcription factors [31]. Tup1 in combination with Nrg1 or Rfg1, or Rbf1 alone carries out negative regulation whereas Efg1, Cph1, Tec1, Czf1, Hgc1, Ume6, Brg1 and Rim101 are involved in positive regulation [75,76]. Efg1 is a chief filamentation regulator under most environmental conditions since it is accountable for the induction of filamentation in response to pathways stimulated by N-Acetyl Glucosamine (GlcNac), pH and CO_2_ [75,77]. A MAPK pathway is required for inducing hyphal growth through Cph1. On the other hand, the signaling pathway involving Dck1 is responsible for the stimulation of filamentation by Czf1, which is triggered by hypoxic conditions [78]. Hgc1 forms a complex with Cdc28 and induces hyphal growth by phosphorylating Efg1 resulting in suppression of cell separation machinery [79]. It is believed that Ume6 drives filamentation through this Hgc1 pathway [80]. The GATA-family transcription factor Brg1 plays a key role in regulating filamentation in *C. albicans* [76].

##### The hunt for small molecules against *C. albicans* filamentation

Researchers have recognized a large number of small molecules tempering with morphogenetic conversions, thereby inhibiting filamentation. Examples include farnesol, phenazine and other autoregulatory alcohols such as retigeric acid and bisbibenzyls, which act as quorum sensing (QS) molecules that target regulators of yeast-to-hyphae transition in *C. albicans* [77–84]. Johnson *et al.* recognized up to 21 different inhibitors of *C. albicans* filamentation using small molecule screening and, consequently, verified that some of these inhibitors were acting through diverse signaling pathways [85,86]. In search of anti-adhesion compounds, Fazly *et al.* [87] screened a series of compounds from the University of Massachusetts Medical School (MA, USA) Small Molecule Facility DIVERset Library (Chembridge), using a high-throughput phenotypic assay, and obtained several bioactive molecules. One of the lead compounds of this study named filastatin, inhibited *Candida* adhesion to human epithelial cells, yeast to hypha transition, biofilm formation and pathogenesis in a nematode infection model. It also inhibited the induction of hyphal specific HWP1 promoter, which is an early and essential event in the process of hyphal development. Zhang *et al*. demonstated that two steroid saponins isolated from *Tribulus terrestris* L., as tigogenin-3-Oβ-D-xylopyranosyl (1–2)-[β-D-xylopyranosyl (1–3)]-β-D glucopyranosyl (1–4)-[alpha-L-rhamnopyranosyl (1–2)]-β-D galactopyranoside ( = TTS-12) and igogenin-3-O-β-D-glucopyranosyl (1–2)-[β-D-xylopyranosyl (1–3)]-β-D-glucopyranosyl (1–4)-β-D-galactopyranoside ( = TTS 15), inhibited hyphal formation in *C. albicans* [88].

To obtain inhibitors of *C. albicans* filamentation, large-scale phenotypic screening of 30,000 drug-like small molecules within ChemBridge's DIVERSet chemical library was performed by Romo *et al.* [89,90]. They obtained several novel bioactive compounds, out of which one main compound with a common biaryl amide core structure was identified as N-[3-(allyloxy)-phenyl]-4-methoxybenzamide. This compound exhibited its antivirulence properties by inhibiting filamentation and biofilm formation in *C. albicans* under *in vitro* and *in vivo* conditions. Pierce *et al.* [91] performed a cell-based phenotypic screening of 2,293 compounds, using three different chemical libraries from the National Cancer Institute's Open Chemical Repository Collection (Naturalset, Structural Diversity set, and Challenge set). They identified 17 confirmed compounds as inhibitors of filamentation in *C. albicans*.

### Adhesion

Adherence of *Candida* to the host tissues or medical devices is a prerequisite for colonization and biofilm formation. Adherence of *Candida* to the host tissues exploit several adhesins, which are expressed on its surface. The hydrophobic proteins entrenched in a matrix of *Candida* cell walls, underneath the fibrillar layer, deliver the hydrophobic interactions required to convert initial interaction between the fungus and the host surface into a strong bond [92]. Adhesins are agglutinin-like sequences and are member of a family of seven glycosylated proteins. The enhanced tissue invasiveness of hyphal cells is attributed to increased adhesiveness due to the expression of agglutinin-like sequence adhesins [31]. The adhesins Als1p, Als3p and Als5p (Ala1p) are located on the cell surface of hyphae and help in adhering to the host's buccal epithelial cells, collagen, endothelial cells, fibronectin and laminin [93]. Als4p, Als6p and Als9p bind to endothelial cells, collagen and laminin, respectively. Als5p is also required for cell aggregation, whereas the role of Als7p is uncertain [31,94]. These adhesins, along with HWP1 and EAP1, are known to mediate biofilm formation of *C. albicans* into the abiotic surfaces or epithelial cells [95,96].

### Biofilms

Biofilms are controlled assemblies of microbial communities onto biotic or abiotic planes, in which the phenotypic behavior of cells, including growth rate and gene expression, is changed compared with the planktonic cells [97]. In *Candida* biofilms, the basal layer is formed by the adherent yeast cells and invasive hyphal forms construct an upper layer above it. The layers are surrounded by a self-produced extracellular polymer matrix of chitins, eDNA, polysaccharides and proteins, which forms a 3D structure with water channels [95,98,99]. A wide array of medical implants such as catheters, endotracheal tubes, pacemakers and other prosthetic devices are easily colonized by biofilm forming fungi, resulting in persistant fungal infections.

*C. albicans* remains the most commonly allied species in this context and exhibits predominant prevalence in nosocomial infections. The majority of clinical manifestations of candidiasis are linked to biofilm formation on such devices. These devices, in turn, not only provide a platform for candidal cells to form a biofilm, but also promote dissemination through the host defense [29,30,100]. Many mucosal-associated diseases such as oropharyngeal candidiasis, denture stomatitis and vaginal candidiasis, are also linked to the ability of *C. albicans* to form biofilms [100]. Similar to filamentation, biofilm development is also QS controlled and is regulated at the molecular level by a complex network. The two molecules – farnesol and tyrosol – regulate the development of biofilms via QS regulated gene expression [99,101–103]. This cell to cell communication regulates overpopulation, nutritional competition and facilitates dissemination of old biofilm cells to establish infection at a distal site [104]. Therefore, biofilm cells are more resistant to eradication, and results in long lasting persistant infections [105].

#### Transcription factors regulating biofilm formation

There are six transcriptional factors regulating biofilm development in *C. albicans* of which Efg1 and Bcr1 are the most studied [103]. The biofilm structure in *C. albicans* is stabilized by hyphal development, which is controlled by a regulatory network of gene *BCR1* [106]. In addition, as *efg1* is the main regulator of morphogenesis and metabolism, it regulates biofilm formation and pathogenesis [107]. Many hypha-specific genes, such as *als1*, *als3* and *hwp1* are controlled by Bcr1 and Efg1 [108,109]. Therefore, *bcr1*, *efg1* and genes under their control may provide new drug targets for developing antipathogenic drugs. One of the *bcr1*-dependent genes is *ece1*, which is responsible for hypha induction in *C. albicans* [110]. Researchers have found that *ece1* regulates adhesion as it is indicated by the fact that overexpression of *ece1* restores biofilm formation in *bcr1/bcr1* mutant strains [108,109]. Therefore, it has become a perfect target for antifungal drug discovery.

### Antibiofilm

Biofilm-associated infections of *Candida* cells can be treated in two ways; by inhibition of biofilm formation and eradication of preformed biofilms. To combat device-related biofilm infections, a strategy is required to restrict the dispersion of biofilm cells. As cells dispersed from the biofilms are responsible for dissemination, extravasation and establishment of invasive candidiasis [105]. The biofilm matrix can also be a target for development of antipathogenic drugs. Extracellular DNA is a key component of the *C. albicans* biofilm matrix and it has been discovered that a combination of DNase with some antifungal drugs improves their efficacies [111]. Since filamentation and biofilm development are coregulated in *C. albicans*, anti-morphogenetic drugs could also potentially retard the development of biofilms. Similar justification stands for the use of QS modulators [12]. However, the strategies may differ for the development of biomaterials resisting *C. albicans* biofilm growth, such as catheter coatings and lock solutions [112,113].

#### Targeting biofilm development in *C. albicans*

Preformed biofilms are usually targeted through various approaches, depending on the nature of the host-candida interaction. However, in many cases, a strategy is adapted to inhibit the formation of biofilm by *Candida* cells [100]. Currently, to overcome the problem of biofilm-associated drug resistance, researchers have exploited calcineurin inhibitors such as cyclosporine A (CsA), tacrolimus (FK506) and heat shock protein 90 (Hsp90) inhibitors for example, geldanamycin [114,115].

Calcineurin is a Ca^2+^-calmodulin-activated phosphatase that regulates intracellular calcium homeostasis; cell cycle progression; morphogenesis; mating and cytokinesis; recovery from pheromone arrest; cell wall biosynthesis; antifungal drug resistance; and pathogenesis [115,116]. Combinations of calcium and calcineurin inhibitors with known antifungal compounds have been shown to inhibit the growth of drug-resistant fungal strains [116–118]. Therefore, inhibition of calcineurin signaling is a novel antifungal strategy, that both attenuates fungal virulence and increases the efficacy of the existing antifungals with concomitant suppression of antifungal resistance [119]. Under *in vitro* and *in vivo* conditions, the planktonic and biofilm cells of *Candida* have been reported to be sensitive to the combination of fluconazole with FK506 or with CsA [115]. A study by Chen *et al.* [120] reported both *in vitro* and *in vivo* synergistic interaction of posaconazole with FK506, against drug-susceptible or resistant *C. albicans* strains. Moreover, Hsp90 inhibitors have exhibited potential synergistic antifungal activity in combination with azoles and echinocandins against the *C. albicans* [121].

Hsp90 is an essential and highly conserved molecular chaperone that facilitates folding, assembly and maturation of proteins in eukaryotes. It has demonstrated its role in biofilm formation and the evolution of drug resistance in *C. albicans* [114,122]. In *C. albicans*, Hsp90 regulates azole resistance through its key downstream effectors, calcineurin and the Mkc1 kinase [123]. Hsp90 stabilizes calcineurin by direct interactions, therefore, the inhibition of Hsp90 is expected to result in depletion or inactivity of the client protein calcineurin [124]. Moreover, Hsp90 inhibitors such as geldanamycin and its derivatives, nongeldanamycin, have exhibited a potential synergistic antifungal activity in combination with azoles or echinocandins against the *C. albicans* [121,125]. It has been observed that compromising Hsp90 function in *C. albicans* resulted in the abrogation of resistance of biofilms to the azoles. The reduction of Hsp90 levels led to a marked decrease in matrix glucan levels, providing a compelling mechanism through which, Hsp90 might regulate biofilm azole resistance. In a rat venous catheter infection model, the weakening of Hsp90 function either genetically or pharmacologically could transform ineffective fluconazole into highly effective drugs in eradicating biofilms [114]. Cowen *et al.* [126] demonstrated the benefit of combining the fluconazole with clinically relevant Hsp90 inhibitors that are structurally related to the natural product geldanamycin (GdA). Using the *Galleria mellonella* model for pathogenesis, they demonstrated synergistic effects between GdA and echinocandin against *Aspergillus fumigatus*. Whereas in a murine model of *C. albicans* disseminated candidiaisis, genetical compromise with HsP90 expression resulted in enhanced activity of fluconazole. Li *et al.* [125] explored the effect of non-Geldanamycin Hsp90 inhibitor molecule HSP990 on the activity of fluconazole against *C. albicans.* They observed that the efficacy of fluconazole against biofilm formation *in vitro* and in a murine model of disseminated candidiasis, is significantly enhanced when used in combination with HSP990. These combination therapies could provide less toxic treatment to the patient and more effective killing of pathogens in a wider range of *Candida* spp.

The high-throughput screening of small molecule compounds from different chemical libraries [127], along with the evaluation of a variety of natural products for anti-biofilm activity [128] could lead to the development of newer alternative therapeutics. To identify compounds inhibiting *C. albicans* biofilm, Pierce *et al.* [129] conducted a large-scale whole-cell assay screen of 20,000 small molecules from the research-intensive and medicinally relevant NOVACore chemical library (Chembridge, CA, USA). They recognized a novel hit series of diazaspiro-decane structural analogs. Compound 61894700 was extensively characterized and displayed potent inhibitory activity against filamentation as well as biofilm in *C. albicans*.

In a study, a high-throughput microarray-based technology was utilized to investigate the biofilm formation in *C. albicans*. They used *Ca*BChip (*Candida albicans* Biofilm Chip), comprised of 768 spatially discrete and equivalent nano-biofilms on a standard microscope glass slide. Although nanoscale biofilms were miniaturized as 2000-fold, *Ca*BChip displayed phenotypic properties such as morphological, architectural and increased drug resistance analogous to biofilm cells formed using conventional 96-well microtiter plate. This automated nanobiofilm chip is easy to handle and is fully compatible with standard microarray technology and equipment [130]. Thus, the use of small-molecule compounds alone or in combination with existing antifungal drugs may provide a potential therapeutic strategy for fungal infectious disease. The use of such technology will encourage the antifungal drug discovery program since it will allow an inexpensive approach that is convenient and rapid in the screening of hundreds-to-thousands of compounds concurrently [12].

### Transcription factors as unique antifungal drug targets

Transcription factors (TFs) are attractive as novel antifungal drug targets since they are evolutionarily divergent between fungi and humans and can be exploited as selective drug targets. Several natural or synthetic or peptidomimetic compounds have been identified that interfere with TFs which regulate many vital gene expressions. These compounds can inhibit hetero- or homodimerization of TFs, TF-binding DNA elements, DNA-binding domains of TFs, or the interaction between a TF and its essential modulating proteins [131]. The functional characterization of fungal TFs and their role in pathogenicity has become a demanding area of research work. Fungal TFs have been analyzed using functional genomic analyses at large-scale in *C. albicans* and *C. neoformans*. In this regard, Nobile and Mitchell [132] generated 83 TF mutants of *C. albicans* and addressed their roles in biofilm formation. Homann *et al*. have characterized *in vitro* functions of 166 TFs under 50 different growth conditions [133]. Furthermore, the comparative functional analysis of TFs in *C. albicans* and *C. neoformans* has provided an insight into the kinds of TFs being exploited as drug targets of broad or narrow spectrum action. The TFs Crz1, Nrg1, Rim101, Bcr1/Usv101, Zap1/Zap104 and Brg1/Gat201, are involved in virulence processes of both of these pathogens. Crz1 directs down-stream target that modulates ion homeostasis, pH response, thermo-tolerance, cell wall integrity, developmental processes and many other different virulence factors in *C. albicans, C. neoformans* and *A. fumigatus* [134].

Considering the industrial perspectives, broad-spectrum antifungals are commercially more rewarding. However, these drugs have associated potential hindrance to be developed as a successful candidate because such drugs disturb normal commensal microflora of the host and cause secondary infections of undesirable pathogens. In fact, *C. albicans* inhabit the gastrointestinal tract of humans as a normal microflora. Therefore, the advantage of this approach is that if the disease-causing specific fungal pathogen is determined in an early stage of infection, the pathogen-specific narrow-spectrum targets could be developed. Any drugs targeting such TFs would facilitate drug discovery to obtain more optimal drugs, reducing the toxic effects. In *C. albicans*, out of many narrow-spectrum TFs targeting agents, Efg1 is best characterized. Targeting of Efg1 also enhances the susceptibility of *Candida* to azole drugs [135,136].

### Metabolic pathways controlling virulence factors

#### Glyoxylate cycle

The glyoxylate cycle is a modification of the tricarboxylic acid cycle, in which the steps for the production of CO_2_ are bypassed, resulting in carbons to be reserved as substrates for gluconeogenesis. In microorganisms this cycle is essential for the uptake and utilization of nonfermentable carbon sources, such as ethanol, acetate and fatty acids [137]. Since this metabolic pathway enables *C. albicans* to survive in a nutrient-restricted environment of the host, it is an essential virulence factor of *C. albicans* [137,138]. Isocitrate lyase (ICL) and maltate synthase are the key enzymes involved in the Glyoxylate cycle. During host infection, pathogenic microorganisms such as *Aspergillus fumigatus, Magnaporthe grisea, Burkholderia pseudomallei, Mycobacterium tuberculosis* and *C. albicans* cause upregulation of the glyoxylate cycle [139,140]. In particular, the *icl* gene of *C. albicans* is strongly activated when cells are exposed to macrophages [137]. Research has shown that disruption of *icl* gene rendered *C. albicans* unable to utilise acetate, ethanol or oleic acid. It has also been found that ICL is crucial for the survival of *C. albicans* in response to macrophage engulfment [137–139].

As this cycle does not exist in mammalian cells, ICL appears to be a prospective target for the development of antifungal drugs [127,138–140]. Several inhibitors of ICL, including 3-nitropropionate, 3-bromopyruvate, 3-phosphoglycerate, mycenon, oxalate and itaconate, have been identified [141]. However, most of these inhibitors are not pharmacologically suitable for use *in vivo* due to their toxicity and nonspecificity. It is expected that inhibitors of specific ICL show lower toxicity [140]. Thus, natural specific inhibitors of ICL derived from organisms have been sought as they may have many suitable pharmacological properties [142]. Bae *et al.* [143] isolated two compounds, mohangamide A and mohangamide B, from a marine actinomycete *Streptomyces* spp. that have shown specific inhibitory activity against the ICL of *C. albicans*. Further development of selective ICL inhibitors with suitable pharmacological properties would require more tests in animal models to establish both the importance of the glyoxylate cycle in *C. albicans* and the evidence for the therapeutic potential of ICL inhibitors in fungal infections [140].

#### High osmolarity glycerol pathway

Responding and adapting to different microenvironments within a host is advantageous for pathogens. The MAPK pathway is considered an important signal network in eukaryotes as it allows adaptation to changes in the environment. Four MAPK signalling pathways in *C. albicans* have been recognized as Mkc1, Cek1, Cek2 and the high osmolarity glycerol (HOG) pathway [144]. These pathways in *C. albicans* are exploited in the biogenesis of cell wall, morphogenesis and stress response [145,146]. Out of these four, the HOG pathway is the choice of the researcher to target in *C. albicans* [147]. It is comprised of a two-component system having a phosphorelay system and Hog1-type MAPK cascade. Most importantly, this two-component-system is conserved in organisms ranging from bacteria to higher plants, but not in mammals [144,147]. Therefore, it can be considered as a promising molecular target for developing new antifungal agents with lesser or no toxicity to the host [140].

#### Target of Rapamycin signaling pathway

The target of rapamycin (TOR) is a member of the phosphoinositide 3-kinase-related protein kinase family and regulates normal physiology and growth in eukaryotes [148,149]. Many studies have shown its contribution in regulating virulence in *C. albicans* [150,151]. The Tco89 sequence homolog, as a member of the TORC1 complex in some fungi and does not exist in mammals. Therefore, it could be a perfect antifungal target [152].

#### Cell calcium homeostasis

Calcium homeostasis in the fungal cell is required for survival, pathogenicity and is responsible for maintaining many closely associated physiological processes in *C. albicans* such as adhesion, hyphal development and stress response [153]. The calcium cell survival pathway in *C. albicans* facilitates the survival of cells in stress conditions of the environment. It elicits Ca^2+^ influx through the Cch1-Mid1 channel and triggers calcineurin and its downstream transcription factor Crz1p [154]. Calcineurin is a calcium-regulated signaling enzyme and needed by *C. albicans* to survive in the serum, therefore, it is also a promising virulence target [155]. Consequently, transcription factor Crz1p is now considered as a newly discovered calcineurin target in *C. albicans* [119].

Calmodulin is a small and universal Ca^2+^ binding protein that is highly conserved in all eukaryotes. It plays an important role in mediating calcium cell survival and response to other stressors in *C. albicans*. Thus, all these transcription factors including Cch1-Mid1 channel, calmodulin, calcineurin, Crz1p and the other components, which are involved in calcium homeostasis, could serve as potential drug targets for developing newer antifungals against *C. albicans* [140,119].

#### QS molecules

Farnesol, a lipid signaling molecule, regulates the invasiveness of hyphae in *C. albicans* by affecting the expression of genes involved in hyphal development [82]. Research has shown that farnesol produced *in situ* by planktonic *C. albicans* cultures prevented biofilm formation. Also, it has been found that the accumulation of farnesol blocks the morphological shift from yeast to hyphae form at high cell densities [101]. A study conducted by Decanis *et al.* [156] suggest that farnesol may reduce *Candida* pathogenesis through a downregulation of yeast-to-hypha morphogenesis, which may involve a modulation of *SAPS* gene expression. The study by Navarathna *et al.* [157] also supports the view of using farnesol as a new antifungal agent. Since *C. albicans* synthesizes farnesol from farnesyl pyrophosphate (FPP), and FPP is the biosynthetic precursor of both farnesol and ergosterol [158]. They hypothesized that drugs blocking the sterol biosynthetic pathway after FPP might lead to the accumulation of FPP, which, in turn, could lead to enhanced farnesol production. This hypothesis proved to be correct as they found that the treatment of mouse model of disseminated candidiasis with subinhibitory concentrations of fluconazole has resulted in a several-fold increase in production of farnesol by treated *Candida* cells compared with untreated cells. The increased production of farnesol inhibits hyphal morphogenesis in *Candida* cells and, it explains the mechanism of antimicrobial action of farnesol [101]. Therefore, farnesol could be considered as a potential candidate to develop antipathogenic antfungal drug against candidiasis.

### Antimetabolic pathways

A lot of research has been conducted so far to target metabolic enzymes to develop anti-candidal agents. A study has revealed that tacrolimus (FK 506) and cyclosporin A isolated from *Streptomyces tsukubaensis* and *Tolypocladium inflatum* Gams, respectively, can hinder virulence factor, calcineurin. Their mechanism of action includes the formation of a complex with intracellular proteins named immunophilins, which are present in fungi as well as in humans [159]. Another study identified some ICL inhibitors such as 3-nitropropionate, mycenon, 3-bromopyruvate, oxalate, 3-phosphoglycerate and itaconate [141].

Recently, mohangamide A and mohangamide B possessing inhibitory activity against ICL of *C. albicans*, were isolated from a marine actinomycete *Streptomyces*, which has led to the designing of new ICL inhibitors [143]. Sato *et al*. [160] confirmed that hyphal growth in *C. albicans* is suppressed by the calmodulin-specific inhibitors, trifluoperazine (TFP) and W–7 by averting the expression of hypha-specific mRNAs. Also, verapamil, a calcium channel blocker has been reported to exert inhibitory effects on adherence, colonization, development of hyphae and biofilm formation in *C. albicans* [153,161].

## Use of molecular approaches to identify new targets

**Figure 2.**
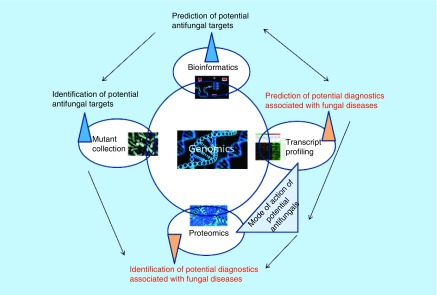
Contribution of genomics in developing antifungals and diagnostics.

With the introduction of microbial genome sequencing of relevant pathogens, greater insights of virulence can be examined. It has allowed the conventional whole-cell-based approach to be replaced by novel target-based drug discovery [35]. Indeed, the availability of genome sequence data for *Candida* spp. has assisted researchers in recognizing novel and potential antifungal targets using bioinformatics approaches including mutant collection, transcript profiling and proteomics. However, the newly explored drug targets using bioinformatic approaches have not yielded any clinically valuable antifungal drugs [162]. Genomics has been proven beneficial in developing novel antifungal agents and new diagnostic tools in various ways, as depicted in Figure 2. The functional genomics is quite useful in serving as a functional tool to explain the emergence of drug-resistance and molecular mechanisms of drugs. The strategic point regarding genomic screens is that it delivers comparatively unbiased visions of the drugs affecting the fungal cell. Bioinformatics approaches have played a successful role in combination therapies; as an example, by predicting the use of one drug targeting a particular process in the fungal cell to be combined synergistically with the second drug inhibiting mechanisms of resistance to the first drug [162,163]. To identify and validate the antifungal drug targets, researchers mainly employ three genetics based molecular tools; gene expression profiling [164–166]; RNA mediated gene silencing [167–169]; and insertional mutagenesis [170–172].

An important approach is to analyze the gene expression profile at the level of transcriptome and proteome to assess the effects of the drugs. In *C. albicans*, transcript profiling has revealed the effects of ketoconazole interfering with the expression of genes involved in lipid, fatty acid and sterol metabolism and, caspofungin, influencing the expression of genes involved in cell wall biosynthesis [173,174]. A variety of RNA-mediated gene silencing or knockout methods have been identified such as the introduction of antisense RNA, double-stranded RNA (dsRNA) (also known as RNA interference or RNAi) and sense transgenes (also known as cosuppression in plants or quelling in fungi). These processes are generally termed as post-transcriptional gene silencing, and have proven their applications in the clinically important fungal human pathogen *C. albicans* [167]. In a study conducted by Disney *et al.* [169], it was found that an antisense DNA oligonucleotide mimicking rRNA was easily taken up by the cell and it could inhibit the growth of a *C. albicans* strain below pH 4.0. Therefore, it is expected that the use of oligonucleotide silencing methods could be a promising approach in antifungal drug discovery. On the other hand, for the creation of homozygous insertion mutants in the diploid *C. albicans*, a new gene disruption cassette, UAU1 marker cassette has been described [170]. The UAU1 marker cassette can be incorporated into a Tn7 transposon that allows the generation of random homozygous insertion mutants in *C. albicans* by *in vitro* transposition [171]. In general, the Tn7-UAU1 approach facilitates a large-scale first-pass assessment of the essentiality of genes and fast phenotypic (e.g., filamentous growth) screening for gene insertions of interest. Whereas, Haselbeck *et al.* [172] reported on a gene replacement and conditional expression method for genome-wide gene identification in *C. albicans*. This approach could also lead to the construction of fungal strains carrying gene fusions or promoter replacements. Additionally, nucleotide biosynthesis could be considered as a suitable antifungal drug target. Flucytosine as such is not active as an antifungal but once metabolized by the cell; it rapidly converts into 5-fluorouracil and blocks the synthesis of DNA and proteins by inhibiting cytosine deaminase, which is absent in human hosts [28].

Overall, the experimental power of genomics has significantly improved the diagnosis and therapeutics in antifungal drug discovery. More could be expected through the wider use of genome-wide profiling and mutant collections and in combination with well-targeted, clinically relevant molecular and cellular approaches.

## Conclusion

So far, several virulence factors in *Candida* have been identified, but many more are yet to be discovered with the advancements of molecular approaches. Interestingly, these determinants are unique to fungi. In the antifungal drug discovery programme, the options of novel potential targets will be widened as more newer virulence factors will be discovered. As hydrolytic enzymes are responsible for increasing the pathogenicity in *Candida* spp., a greater effort should be made in understanding their roles in fungal pathogens to develop antipathogenic drugs. Moreover, because of its prime role during disease progression, the morphogenetic conversion in *C. albicans* has been most studied by researchers. However, the knowledge of filamentation and biofilm formation in *C. albicans* at the molecular level has been quite augmented, but until now it has not been possible to harness these targets for the development of new drugs for candidiasis. In search of filamentation and biofilms specific inhibitors, the accumulation of information along with the implementation of high-throughput screenings might provide much needed newer compounds to the antifungal armamentarium. Studies have yielded unique insights into the regulatory metabolic circuits and transcription factors that control different virulence functions associated with these processes and have led to the identification of small-molecule inhibitors. Indeed, an increasing number of small molecules are being discovered that can modulate morphogenetic conversions and prevent hyphal or biofilm development in *C. albicans*. Future investigations into precise targets of virulence processes as mentioned in this article are needed to understand the fundamental mechanisms of pathogenicity in *C. albicans* and to facilitate the antifungal drug discovery.

## Future perspective

The viability of the antipathogenic drug development approach is due to the recent advancements in infection biology and bioinformatics. In the search for future antifungals, investigations of antivirulence compounds, target validation and preliminary screenings are being performed by various researchers to identify potential drug candidates. However, none of these compounds with the proposed newer mechanism of action have yet been introduced in clinical settings. It is still undecided if these approaches are or will be valuable to our expectations or not. We keenly await novel research in the next few years that could shed light on the performance of such antifungals, their ability to reduce resistance development and whether they could potentiate the efficacy of current antifungals in combination or would have the ability to make existing antifungals obsolete.

**Executive summary**

Currently available antifungal drugs are ineffective in controlling candidiasis:

- Despite the existing armamentarium of antifungal drugs such as azoles and amphotericin B, the rate of morbidity and mortality associated with candidiasis has been increasing.
- The emergence of drug resistance and host toxicity is a major hindrance to the success of these drugs.

There is a need for alternative strategies to employ to discover newer antifungal agents:

- Targeting virulence and biofilm formation in *Candida* could be a fruitful strategy to develop newer antifungal drugs.
- Various virulence factors have been recognized in fungi, especially in *C. albicans*, such as the production of extracellular hydrolytic enzymes such as proteinase, phospholipase, hemolysin, adhesion, morphogenesis and biofilm formation.

These virulence factors could be controlled by regulating their production at a molecular or metabolic level:

- Several research efforts have envisaged the antipathogenic drugs that inhibit the production of these virulence factors.
- Screening of small molecules using high-throughput sequencing has further led to the discovery of many compounds that target one or more virulence factors in fungi.

The successful use of antipathogenic drugs has not yet been reported in clinical practice:

- Ongoing research equipped with advancements of genomics and proteomics in studying host–pathogen relationships could reveal the success or failure of this strategy in the near future.
